# Supplementary material for: Multi‐dataset identification of innovative feature genes and molecular mechanisms in keratoconus
Source: J Cell Mol Med. 2024 Sep 19;28(18):e70079. doi: 10.1111/jcmm.70079 (PMC11412914; doi:10.1111/jcmm.70079)
Supplement: Supplementary file 2 — Material S2. [file JCMM-28-e70079-s002.zip › 7.GSEA analysis/2.ATOH7/KEGG_AMINO_SUGAR_AND_NUCLEOTIDE_SUGAR_METABOLISM.html]

Details for gene set KEGG\_AMINO\_SUGAR\_AND\_NUCLEOTIDE\_SUGAR\_METABOLISM[GSEA]

|  || Dataset | ATOH7.group.cls#h\_versus\_l |
| Phenotype | group.cls#h\_versus\_l |
| Upregulated in class | h |
| GeneSet | KEGG\_AMINO\_SUGAR\_AND\_NUCLEOTIDE\_SUGAR\_METABOLISM |
| Enrichment Score (ES) | 0.413071 |
| Normalized Enrichment Score (NES) | 1.4801302 |
| Nominal p-value | 0.09917355 |
| FDR q-value | 0.27004975 |
| FWER p-Value | 0.813 |
Table: GSEA Results Summary

  

Fig 1: Enrichment plot: KEGG\_AMINO\_SUGAR\_AND\_NUCLEOTIDE\_SUGAR\_METABOLISM      
 Profile of the Running ES Score & Positions of GeneSet Members on the Rank Ordered List

  

| SYMBOL | TITLE | RANK IN GENE LIST | RANK METRIC SCORE | RUNNING ES | CORE ENRICHMENT || 1 | CHIA | na | 123 | 0.747 | 0.2191 | Yes |
| 2 | GCK | na | 1752 | 0.364 | 0.2807 | Yes |
| 3 | UAP1 | na | 5667 | 0.180 | 0.2216 | Yes |
| 4 | HK2 | na | 5782 | 0.177 | 0.2711 | Yes |
| 5 | GMPPB | na | 6913 | 0.145 | 0.2817 | Yes |
| 6 | GFPT2 | na | 7391 | 0.133 | 0.3076 | Yes |
| 7 | GMPPA | na | 8757 | 0.103 | 0.2990 | Yes |
| 8 | PGM1 | na | 9190 | 0.096 | 0.3150 | Yes |
| 9 | GNPDA2 | na | 9524 | 0.090 | 0.3321 | Yes |
| 10 | PGM3 | na | 9568 | 0.089 | 0.3575 | Yes |
| 11 | PMM1 | na | 9721 | 0.086 | 0.3786 | Yes |
| 12 | GFPT1 | na | 9726 | 0.086 | 0.4041 | Yes |
| 13 | GPI | na | 10220 | 0.078 | 0.4131 | Yes |
| 14 | HK1 | na | 11153 | 0.064 | 0.4052 | No |
| 15 | UGDH | na | 12373 | 0.048 | 0.3843 | No |
| 16 | GALT | na | 12980 | 0.041 | 0.3789 | No |
| 17 | CYB5R1 | na | 12999 | 0.040 | 0.3905 | No |
| 18 | HK3 | na | 13130 | 0.039 | 0.3984 | No |
| 19 | RENBP | na | 14767 | 0.023 | 0.3581 | No |
| 20 | UGP2 | na | 14844 | 0.023 | 0.3626 | No |
| 21 | HEXB | na | 14910 | 0.022 | 0.3673 | No |
| 22 | GMDS | na | 15093 | 0.020 | 0.3681 | No |
| 23 | UXS1 | na | 15701 | 0.015 | 0.3551 | No |
| 24 | HEXA | na | 16012 | 0.013 | 0.3499 | No |
| 25 | NPL | na | 16721 | 0.007 | 0.3316 | No |
| 26 | GNE | na | 17154 | 0.003 | 0.3201 | No |
| 27 | CHIT1 | na | 17327 | 0.002 | 0.3156 | No |
| 28 | CYB5R3 | na | 17761 | -0.002 | 0.3037 | No |
| 29 | GALK1 | na | 17844 | -0.003 | 0.3021 | No |
| 30 | MPI | na | 18144 | -0.005 | 0.2950 | No |
| 31 | PGM2 | na | 18277 | -0.006 | 0.2931 | No |
| 32 | NANS | na | 18906 | -0.012 | 0.2786 | No |
| 33 | GNPNAT1 | na | 19270 | -0.015 | 0.2727 | No |
| 34 | NAGK | na | 20268 | -0.024 | 0.2512 | No |
| 35 | GALK2 | na | 20303 | -0.025 | 0.2576 | No |
| 36 | CMAS | na | 21079 | -0.032 | 0.2449 | No |
| 37 | GNPDA1 | na | 22323 | -0.047 | 0.2230 | No |
| 38 | FPGT | na | 22330 | -0.047 | 0.2367 | No |
| 39 | AMDHD2 | na | 24133 | -0.071 | 0.2060 | No |
| 40 | PMM2 | na | 25623 | -0.097 | 0.1919 | No |
| 41 | NANP | na | 26042 | -0.105 | 0.2111 | No |
| 42 | GALE | na | 27247 | -0.132 | 0.2157 | No |
Table: GSEA details [plain text format]

  

Fig 2: KEGG\_AMINO\_SUGAR\_AND\_NUCLEOTIDE\_SUGAR\_METABOLISM      
 Blue-Pink O' Gram in the Space of the Analyzed GeneSet

  

Fig 3: KEGG\_AMINO\_SUGAR\_AND\_NUCLEOTIDE\_SUGAR\_METABOLISM: Random ES distribution      
 Gene set null distribution of ES for **KEGG\_AMINO\_SUGAR\_AND\_NUCLEOTIDE\_SUGAR\_METABOLISM**
